# Impact of attenuation correction of radiotherapy hardware for positron emission tomography‐magnetic resonance in ano‐rectal radiotherapy patients

**DOI:** 10.1002/acm2.14193

**Published:** 2023-11-03

**Authors:** Jonathan J. Wyatt, George Petrides, Rachel A. Pearson, Hazel M. McCallum, Ross J. Maxwell

**Affiliations:** ^1^ Translational and Clinical Research Institute Newcastle University Newcastle UK; ^2^ Northern Centre for Cancer Care Newcastle upon Tyne Hospitals NHS Foundation Trust Newcastle UK; ^3^ Nuclear Medicine Department Newcastle upon Tyne Hospitals NHS Foundation Trust Newcastle UK

**Keywords:** anal cancer, attenuation correction, PET‐MR, Positron Emission Tomography, radiotherapy, rectal cancer

## Abstract

**Background:**

Positron Emission Tomography‐Magnetic Resonance (PET‐MR) scanners could improve ano‐rectal radiotherapy planning through improved Gross Tumour Volume (GTV) delineation and enabling dose painting strategies using metabolic measurements. This requires accurate quantitative PET images acquired in the radiotherapy treatment position.

**Purpose:**

This study aimed to evaluate the impact on GTV delineation and metabolic parameter measurement of using novel Attenuation Correction (AC) maps that included the radiotherapy flat couch, coil bridge and anterior coil to see if they were necessary.

**Methods:**

Seventeen ano‐rectal radiotherapy patients received a 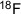
‐FluoroDeoxyGlucose PET‐MR scan in the radiotherapy position. PET images were reconstructed without (${\mathrm{CTAC}}_{\mathrm{std}}$) and with (${\mathrm{CTAC}}_{\mathrm{cba}}$) the radiotherapy hardware included. Both AC maps used the same Computed Tomography image for patient AC. Semi‐manual and threshold GTVs were delineated on both PET images, the volumes compared and the Dice coefficient calculated. Metabolic parameters: Standardized Uptake Values ${\mathrm{SUV}}_{\max}$, ${\mathrm{SUV}}_{\mathrm{mean}}$ and Total Lesion Glycolysis (TLG) were compared using paired *t*‐tests with a Bonferroni corrected significance level of p=0.05/8=0.006.

**Results:**

Differences in semi‐manual GTV volumes between ${\mathrm{CTAC}}_{\mathrm{cba}}$ and ${\mathrm{CTAC}}_{\mathrm{std}}$ were approaching statistical significance (difference −15.9%±1.6%, p=0.007), with larger differences in low FDG‐avid tumours (${\mathrm{SUV}}_{\mathrm{mean}}<8.5\;g\;{\mathrm{mL}}^{-1}$). The ${\mathrm{CTAC}}_{\mathrm{cba}}$ and ${\mathrm{CTAC}}_{\mathrm{std}}$ GTVs were concordant with Dice coefficients 0.89±0.01 (manual) and 0.98±0.00 (threshold). Metabolic parameters were significantly different, with ${\mathrm{SUV}}_{\max}$, ${\mathrm{SUV}}_{\mathrm{mean}}$ and TLG differences of −11.5%±0.3% (p<0.001), −11.6%±0.3% (p<0.001) and −13.7%±0.6% (p=0.003) respectively. The TLG difference resulted in 1/8 rectal cancer patients changing prognosis group, based on literature TLG cut‐offs, when using ${\mathrm{CTAC}}_{\mathrm{cba}}$ rather than ${\mathrm{CTAC}}_{\mathrm{std}}$.

**Conclusions:**

This study suggests that using AC maps with the radiotherapy hardware included is feasible for patient imaging. The impact on tumour delineation was mixed and needs to be evaluated in larger cohorts. However using AC of the radiotherapy hardware is important for situations where accurate metabolic measurements are required, such as dose painting and treatment prognostication.

## 1 INTRODUCTION

Positron Emission Tomography‐Magnetic Resonance (PET‐MR) scanners have great potential for pelvic radiotherapy planning through high‐quality MR anatomical and functional imaging combined with simultaneous PET molecular information.^[^ ^1^ ^]^ This can be used for more accurate delineation of the Gross Tumour Volume (GTV),^[^ ^2^, ^3^ ^]^ delineation of tumour sub‐volumes for radiotherapy dose painting^[^ ^4^ ^]^ and/or as a prognostic tool to identify poorer prognosis patients for dose escalation.^[^ ^5^ ^]^

For anal cancers, 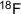
‐FluoroDeoxyGlucose (FDG)‐PET has demonstrated significantly smaller GTVs compared to CT^[^ ^2^ ^]^ and good corresponance with MR.^[^ ^3^ ^]^ A study in rectum cancer patients showed reduced inter‐observer variability for tumour delineations on 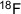
‐FDG‐PET‐CT compared to CT alone.^[^ ^6^ ^]^ PET imaging also has good potential for automatic delineation methods utilising the semiquantitative metric Standard Uptake Value (SUV),^[^ ^7^ ^]^ with automatic methods showing good agreement with manual contours^[^ ^8^ ^]^ and better agreement with pathological analysis than CT or MR.^[^ ^9^ ^]^ PET derived metabolic parameters such as the maximum SUV within a tumour ($SUV_{max}$) and Total Lesion Glycolysis (TLG) have also shown promise as prognostic factors for rectal cancers.^[^ ^5^, ^10^ ^]^

High quality PET imaging is required for accurate GTV delineation and accurate PET SUVs are essential for radiotherapy dose painting and patient prognostics. High quality, quantitative PET imaging requires accurate attenuation correction (AC) of all objects traversed by the annihilation photons.^[^ ^11^ ^]^ However, images used for radiotherapy planning need to be acquired in the radiotherapy position, which requires dedicated radiotherapy hardware such as a flat couch‐top and coil bridges.^[^ ^12^ ^]^ For PET‐MR this is challenging since the radiotherapy hardware will non‐uniformly attenuate the PET signal^[^ ^13^ ^]^ and will not be visible in the MR images. In addition, the flexible anterior MR coil essential for acquiring high quality MR images also has a substantial and non‐uniform PET attenuation.^[^ ^14^ ^]^ Previously, a phantom study has demonstrated a reduction in PET‐MR image quality from acquiring images in the radiotherapy position with a PET activity loss of −17.7%±0.1%, which was greater than the −8.3%±0.2% activity loss caused by the anterior MR coil alone.^[^ ^15^ ^]^ The flexible shape and variable position of the anterior coil makes accounting for it in PET AC maps difficult, so in routine diagnostic use it is ignored.^[^ ^14^ ^]^ However, an advantage of the radiotherapy position is that the use of the coil bridge means the anterior coil shape is fixed and coil position is known. The prior phantom study utilised this to develop a AC map of the radiotherapy hardware and MR anterior coil.^[^ ^15^ ^]^ Evaluation of this method on a phantom demonstrated reduction in the PET activity loss to −2.7%±0.1% compared to phantom measurements made without radiotherapy hardware or anterior coil. The aim of this study was to test the feasibility of using these AC maps in ano‐rectal radiotherapy patients and to determine the impact on GTV delineation and SUV measurements to see if using these AC maps was necessary.

## 2 MATERIALS AND METHODS

### 2.1 Patient data collection

17 patients enrolled in the Deep MR‐only RT study (research ethics committee reference 20/LO/0583) who were planned for radical/neoadjuvant chemoradiotherapy for ano‐rectal cancer and received a PET‐MR scan were included in this sub‐study. Exclusion criteria included contraindications for MR scanning, medical implants in the pelvic area (e.g., hip prostheses) and external contour greater than the scanner field of view. Ten female and seven male patients were included with median age 64 years (range 49–76 years). Patients were diagnosed with rectal cancers (*n* = 8) stages T2N1M0 to T4N2Mx and anal cancers (*n* = 9) stages T1/2N0M0 to T4N3M0.

**FIGURE 1:**
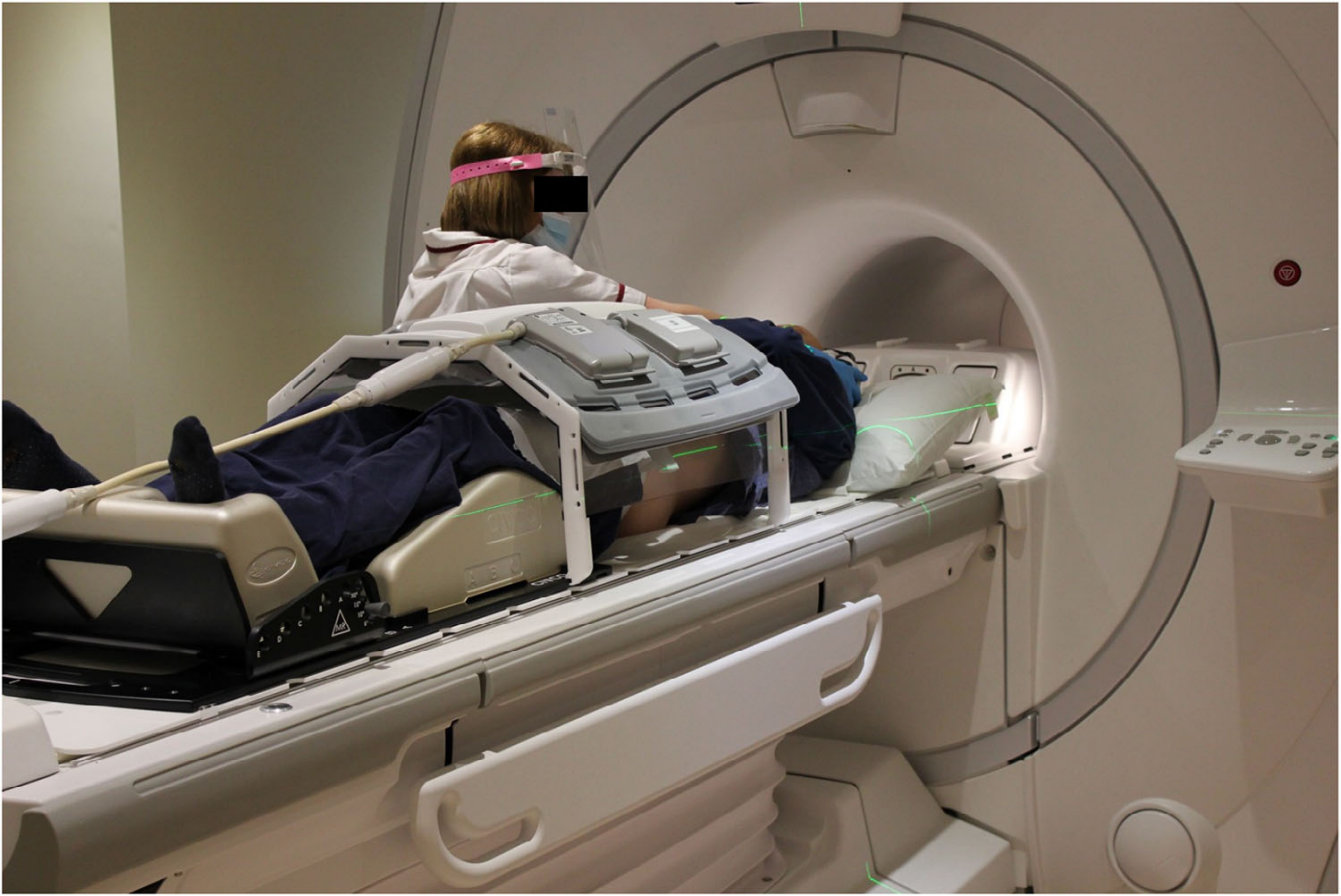
Example of patient setup showing the flat couch top, patient immobilisation device, coil bridge and anterior array coil.

All patients received a simultaneous PET‐MR scan on a SIGNA PET/MR 3T scanner (version MP26 GE Healthcare, Waukesha, USA) after their radiotherapy planning CT scan and before their first treatment fraction. Patients were scanned in the radiotherapy treatment position on a flat couch‐top with a coil bridge for the anterior MR coil as shown in Figure 1.^[^ ^15^ ^]^ Patients were positioned to match their radiotherapy planning CT scan using a combined customisable foot and knee rest (Civco) and external lasers matched to patient tattoos. Immediately prior to entering the scan room patients emptied their bladder and drank 400mL of water. The PET acquisition started 20 min (median, range 15–37 min) after patient drinking. The PET images were acquired 70 min (median, range 60–86 min) after injection with $3.5\;\mathrm{MBq}\;{\mathrm{kg}}^{-1}\pm 10\%$ of 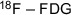 (one patient received $1.7\;\mathrm{MBq}\;{\mathrm{kg}}^{-1}$). All patients had fasted for 6 h prior to injection and had a measured blood glucose concentration of $<10\;\mathrm{mmol}\;L^{-1}$. The PET acquisition consisted of one 5 min bed position with the patient tumour centred in the PET field of view. Images were reconstructed using a Bayesian penalized‐likelihood iterative image reconstruction (Q.Clear) with a relative noise regularizing term factor of β=350 ^[^ ^16^ ^]^ with point spread function correction and time of flight information.

MR images were acquired using the automatic Dixon sequence used for the scanner‐generated PET AC maps. This was a 3D sequence with a voxel size of $2.0\times 2.0\times 2.6\;{\mathrm{mm}}^{3}$ and a field of view $500\times 500\times 312\;{\mathrm{mm}}^{3}$. The images were acquired with a repetition time 4.05ms, echo times 2.232ms (in‐phase) and 1.116ms (out‐phase) and a receive bandwidth of $1302\;{\mathrm{Hzpixel}}^{-1}$. An additional 3D T2‐weighted turbo spin echo sequence was acquired as an anatomical reference for the PET image. This had a voxel size of $1.0\times 1.0\times 2.0\;{\mathrm{mm}}^{3}$, field of view $380\times 304\times 360\;{\mathrm{mm}}^{3}$, repetition time 2000ms, echo time 148ms and a receive bandwidth of $658\;{\mathrm{Hzpixel}}^{-1}$.

All patients received contrast‐enhanced planning CT scans (Sensation Open, Siemens, Erlangen, Germany) in the radiotherapy position. The CT images had a voxel size of $1.1\times 1.1\times 3\;{\mathrm{mm}}^{3}$ and a tube voltage of V=120kVp. Patients were imaged following routine bladder preparation consisting of an empty bladder 30 min prior to the scan, followed by drinking 400ml of water, and bowel preparation consisting of the application of a micro‐enema 60 min prior to the scan followed by bowel emptying.

### 2.2 Attenuation correction maps

AC maps can be divided into two components: a map of the patient and a map of all hardware components within the PET lines or response. For the purposes of this study what was used for the patient map did not matter as long as it was consistent between all PET images. We decided to use the patient CT acquired in the same radiotherapy position as the PET‐MR since CT is the gold standard source of patient AC. The CT was rigidly registered to the in‐phase MR image in RayStation (v9B, RaySearch Laboratories, Stockholm, Sweden). The external contour of the in‐phase MR was automatically delineated using RayStation's function, and manually modified where necessary. The registered CT was cropped to the MR external contour, with any tissue outside the CT external contour but inside the MR external contour set to water density. Any air within the patient was automatically delineated and set to water density.

**FIGURE 2:**
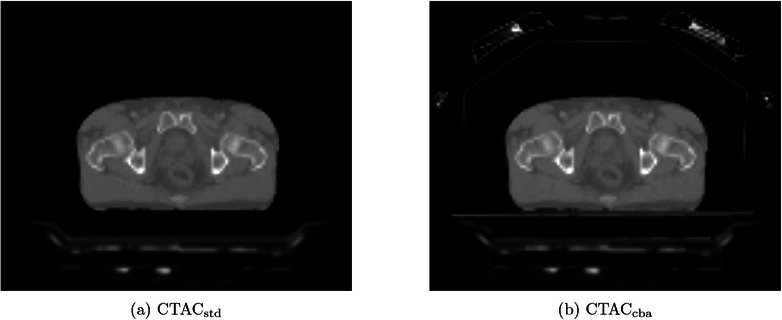
Attenuation correction maps for an example patient.

Two different hardware AC maps were used, each with the CT patient map: ${\mathrm{CTAC}}_{\mathrm{std}}$ and ${\mathrm{CTAC}}_{\mathrm{cba}}$. ${\mathrm{CTAC}}_{\mathrm{std}}$ was automatically generated by the scanner and included the MR spine coil components within the scanner bed. ${\mathrm{CTAC}}_{\mathrm{cba}}$ was the same as ${\mathrm{CTAC}}_{\mathrm{std}}$ but with the manual addition of a model of the radiotherapy couch placed abutting the patient posterior edge and a model of the coil bridge and anterior coil, as described in Wyatt et al.^[^ ^15^ ^]^ The coil bridge and anterior coil model was placed in the patient right‐left and anterior‐posterior directions using the measured distances to the radiotherapy couch. The inferior‐superior position was calculated through landmarking the scanner table to the centre of the coil bridge and using the scanner table position during the PET acquisition, accessible through the private DICOM tag ‘PET_table_z_position’. Examples of the three attenuation maps are shown in Figure 2. ${\mathrm{CTAC}}_{\mathrm{std}}$ would be the hardware AC map produced directly by the scanner without modification whereas ${\mathrm{CTAC}}_{\mathrm{cba}}$ would include all hardware within the PET lines of response. This study aimed to assess whether the improvement in PET accuracy from using ${\mathrm{CTAC}}_{\mathrm{cba}}$ would result in clinically significant differences in GTV delineation and SUV measurements or whether ${\mathrm{CTAC}}_{\mathrm{std}}$ was accurate enough for radiotherapy purposes.

### 2.3 Tumour delineation

**FIGURE 3:**
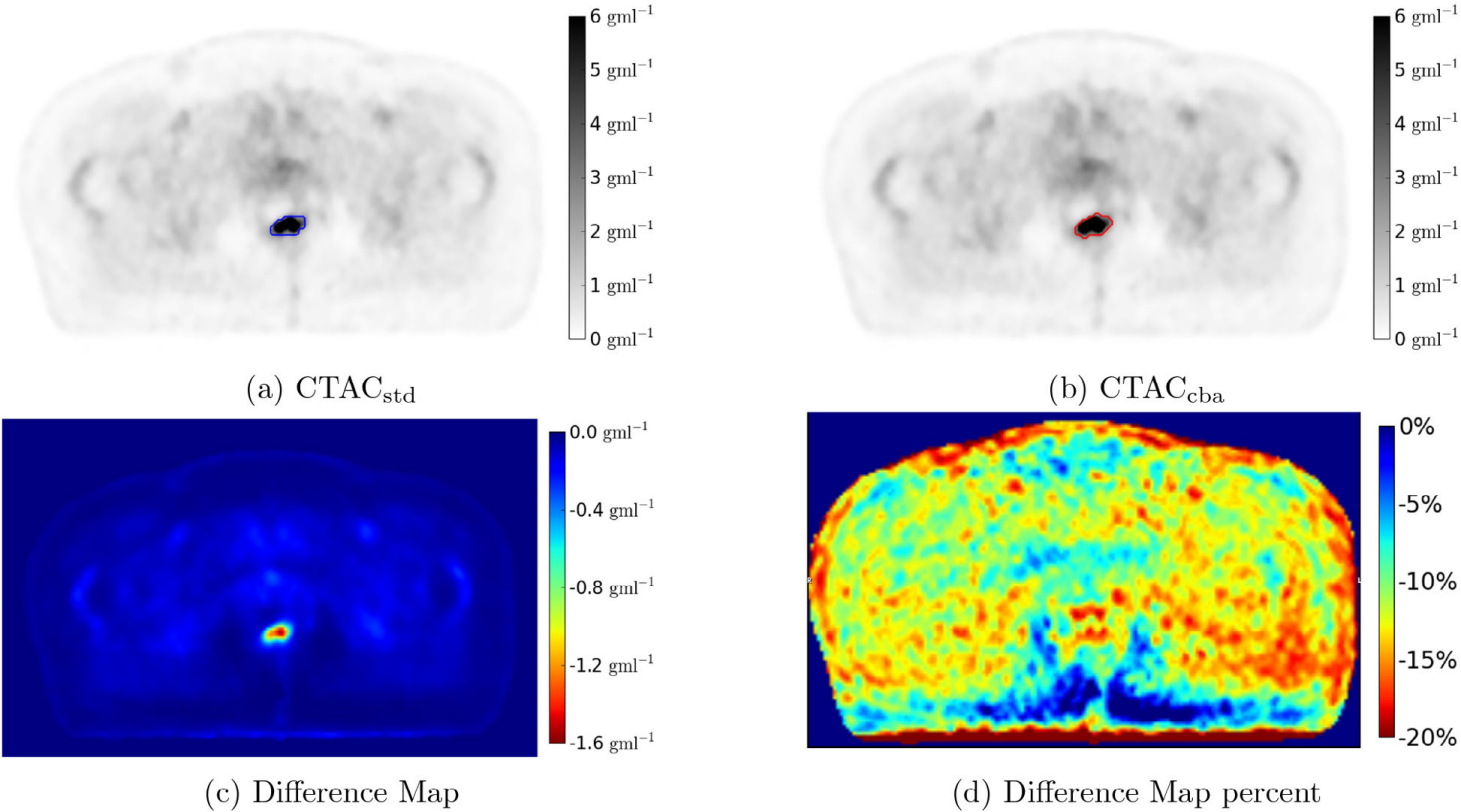
Example PET images reconstructed using the ${\mathrm{CTAC}}_{\mathrm{std}}$ (a) and ${\mathrm{CTAC}}_{\mathrm{cba}}$ (b) attenuation correction maps. The ${\mathrm{GTVp}}_{\mathrm{std}}^{\mathrm{man}}$ (a, blue contour) and ${\mathrm{GTVp}}_{\mathrm{cba}}^{\mathrm{man}}$ (b, red contour) are shown for image respectively. The per‐pixel SUV difference map (${\mathrm{CTAC}}_{\mathrm{std}}$‐${\mathrm{CTAC}}_{\mathrm{cba}}$) is also shown (c) and the per‐pixel percentage difference in (d). PET, positron emission tomography.

The ${\mathrm{CTAC}}_{\mathrm{std}}$ and ${\mathrm{CTAC}}_{\mathrm{cba}}$ PET images were independently contoured at least 7 weeks apart by an experienced consultant PET radiologist using RayStation. The image was automatically thresholded using a fixed $\mathrm{SUV}=2.5\;g\;{\mathrm{mL}}^{-1}$ ^[^ ^8^ ^]^ and the resultant volume manually adjusted by the radiologist as appropriate to represent a gross tumour volume (${\mathrm{GTV}}_{\mathrm{std}}^{\mathrm{man}}$ and ${\mathrm{GTV}}_{\mathrm{cba}}^{\mathrm{man}}$ for the ${\mathrm{CTAC}}_{\mathrm{std}}$ and ${\mathrm{CTAC}}_{\mathrm{cba}}$ images respectively). This was done to reduce intra‐observer variability between the delineations on the two images, with $2.5\;g\;{\mathrm{mL}}^{-1}$ considered diagnostic for malignant tumours in anorectal cancer.^[^ ^8^ ^]^ Primary and nodal volumes were delineated separately (GTVp and GTVn respectively). Examples of the PET images and semi‐manual GTV contours are shown in Figure 3.

A threshold method was also used to automatically delineate the tumour on both ${\mathrm{CTAC}}_{\mathrm{std}}$ and ${\mathrm{CTAC}}_{\mathrm{cba}}$ images, referred to as ${\mathrm{GTV}}_{\mathrm{std}}^{\mathrm{thresh}}$ and ${\mathrm{GTV}}_{\mathrm{cba}}^{\mathrm{thresh}}$ respectively. A threshold value of 40% of the maximum SUV within the manual GTV contour of the relevant image was calculated and voxels with a SUV above that threshold were included in the contour using RayStation.^[^ ^2^ ^]^ The thresholded contour was limited to be within a 0.5cm expansion of the manual GTV contour of the relevant image to ensure physiological uptake was not included, except for patients (*n* = 3) where the GTV abutted the bladder, where a 0.0cm expansion was used in that direction.

### 2.4 Whole image analysis

The per pixel percentage difference in SUV for ${\mathrm{CTAC}}_{\mathrm{std}}$ and ${\mathrm{CTAC}}_{c}$ compared to ${\mathrm{CTAC}}_{\mathrm{cba}}$ were calculated using MICE Toolkit (v1.0.8).^[^ ^17^ ^]^ An external contour was segmented on ${\mathrm{CTAC}}_{\mathrm{cba}}$ using a threshold of $0.05\;g\;{\mathrm{mL}}^{-1}$ and only differences within this external contour were included. A histogram of differences was calculated using 400 bins between −100% and +100% for each patient, and the mean difference within each bin over all patients determined.

The ${\mathrm{CTAC}}_{\mathrm{cba}}$ PET image was used as the reference image for all analyses since a previous phantom study had showed it had the smallest PET activity loss compared to a gold standard PET acquisition without radiotherapy hardware or anterior coil.^[^ ^15^ ^]^ The aim of this study was to assess whether this improvement in SUV accuracy translated into clinically relevant differences in tumour delineation and metabolic parameter measurements.

### 2.5 Tumour delineation analysis

The semi‐manual and thresholded GTV contours were compared between ${\mathrm{CTAC}}_{\mathrm{std}}$ and ${\mathrm{CTAC}}_{\mathrm{cba}}$ to determine the impact on radiotherapy target delineation of not including the radiotherapy hardware with the AC. The contours were compared using the following metrics: the volumetric Dice coefficient, the mean distance to agreement and the GTV volume, all calculated within RayStation. Due to the large variation between patients in GTV volume, the comparisons between ${\mathrm{CTAC}}_{\mathrm{std}}$ and ${\mathrm{CTAC}}_{\mathrm{cba}}$ PET images were performed as per‐patient percentage differences (${\mathrm{CTAC}}_{\mathrm{std}}$ ‐ ${\mathrm{CTAC}}_{\mathrm{cba}}$) relative to the ${\mathrm{CTAC}}_{\mathrm{cba}}$ result. The significance of these differences were evaluated using paired *t*‐tests, with a Bonferroni corrected significance level of p=0.05/(4×2)=0.006, correcting for the four different parameters were being tested (differences in GTV volume, ${\mathrm{SUV}}_{\max}$, ${\mathrm{SUV}}_{\mathrm{mean}}$ and TLG) on both semi‐manual and threshold contours.

### 2.6 Metabolic parameter analysis

The semi‐manual and thresholded GTV contours were compared on metabolic parameters: ${\mathrm{SUV}}_{\max}$, ${\mathrm{SUV}}_{\mathrm{mean}}$ and TLG. TLG was defined as the multiplication of ${\mathrm{SUV}}_{\mathrm{mean}}$ with GTV volume. These would not directly affect tumour delineation using PET, but have shown value as a prognostic factor for rectum patients^[^ ^5^ ^]^ and so would have an impact on dose painting approaches or the personalisation of dose prescriptions based on the PET data. The large variation between patients in values meant the metabolic parameters were also evaluated as per‐patient percentages differences. Statistical significance was assessed using paired t‐tests with the same significance level (p=0.006).

The impact on the prognostic value of PET imaging in the radiotherapy position of using ${\mathrm{CTAC}}_{\mathrm{cba}}$ and ${\mathrm{CTAC}}_{\mathrm{std}}$ was assessed using TLG according to the methods presented in refs. [5, 10]. Literature cut‐off values were only available for rectal cancers so the anal cancer patients were not included in this analysis. The volume used in the TLG calculation was thresholded using either 30% (Ogawa et al.) or 50% (Choi et al.) of ${\mathrm{SUV}}_{\max}$. Although neither study used the 40% of ${\mathrm{SUV}}_{\max}$ threshold used in this study, the thresholds were within 10% which was considered similar enough to apply the cut‐off values. For Ogawa et al. TLG was determined for a combination of primary and nodal disease, whereas for Choi et al. only primary volumes were used. Therefore the TLG for the primary GTVs were compared to 125.84g (Choi et al.) and the combined primary and nodal TLGs to 341g (Ogawa et al.). Patients who changed prognostic groups depending on whether TLG was calculated using ${\mathrm{CTAC}}_{\mathrm{cba}}$ or ${\mathrm{CTAC}}_{\mathrm{std}}$ PET images were recorded.

## 3 RESULTS

### 3.1 Whole image

**FIGURE 4:**
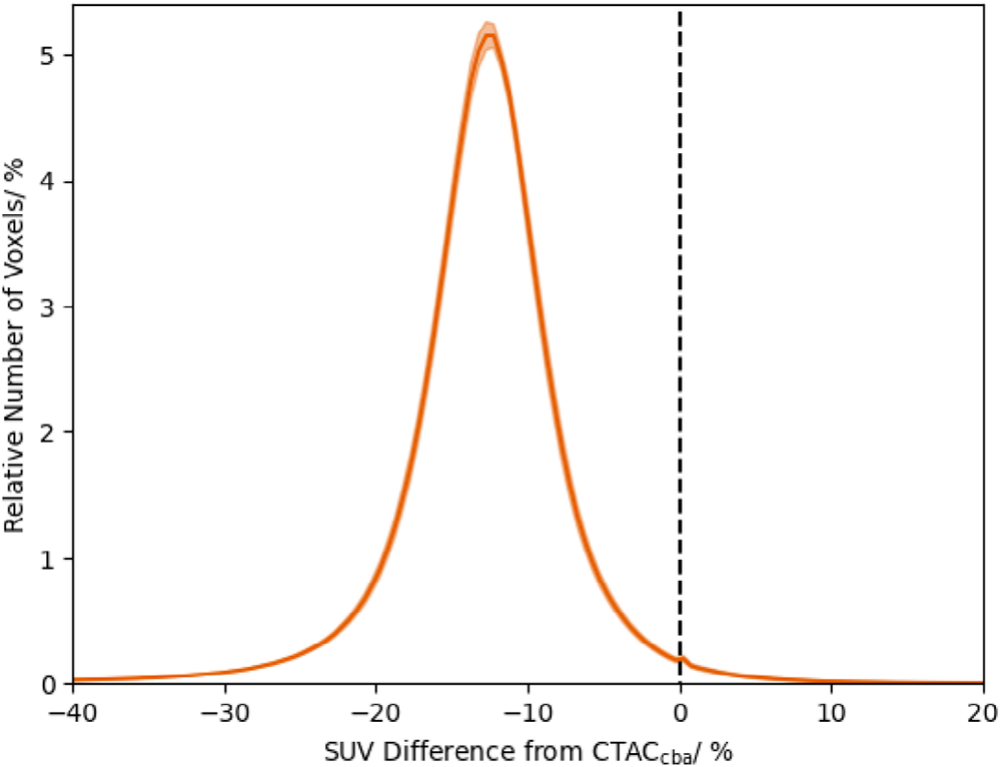
Histogram of number of voxels with percentage differences in SUV between ${\mathrm{CTAC}}_{\mathrm{cba}}$ and ${\mathrm{CTAC}}_{\mathrm{std}}$. The solid line shows the mean counts over all patients for each bin, and shaded areas ± one standard error.

The distribution of SUVs across the image in ${\mathrm{CTAC}}_{\mathrm{std}}$ were lower than ${\mathrm{CTAC}}_{\mathrm{cba}}$, with a mean difference of −13.8%. This is apparent in the histogram plots of differences between ${\mathrm{CTAC}}_{\mathrm{std}}$ to ${\mathrm{CTAC}}_{\mathrm{cba}}$ (Figure 4).

### 3.2 Tumour delineation

Sixteen primary and 10 nodal GTVs were delineated. One patient was being treated post‐surgery and had no primary GTV. The semi‐manual primary GTV volumes were larger than the thresholded volumes, $44.3\pm 14.3\;{\mathrm{cm}}^{3}$ (mean ± standard error, range $2.4\;{\mathrm{cm}}^{3}$, $239.4\;{\mathrm{cm}}^{3}$) and $18.9\pm 5.8\;{\mathrm{cm}}^{3}$ ($0.7\;{\mathrm{cm}}^{3}$, $95.7\;{\mathrm{cm}}^{3}$) respectively. The nodal volumes were more similar, with the semi‐manual volumes being $15.6\pm 6.9\;{\mathrm{cm}}^{3}$ ($0.4\;{\mathrm{cm}}^{3}$, $66.7\;{\mathrm{cm}}^{3}$ ) compared to $7.7\pm 2.7\;{\mathrm{cm}}^{3}$ ($0.8\;{\mathrm{cm}}^{3}$, $22.0\;{\mathrm{cm}}^{3}$) for the thresholded volumes.

**FIGURE 5:**
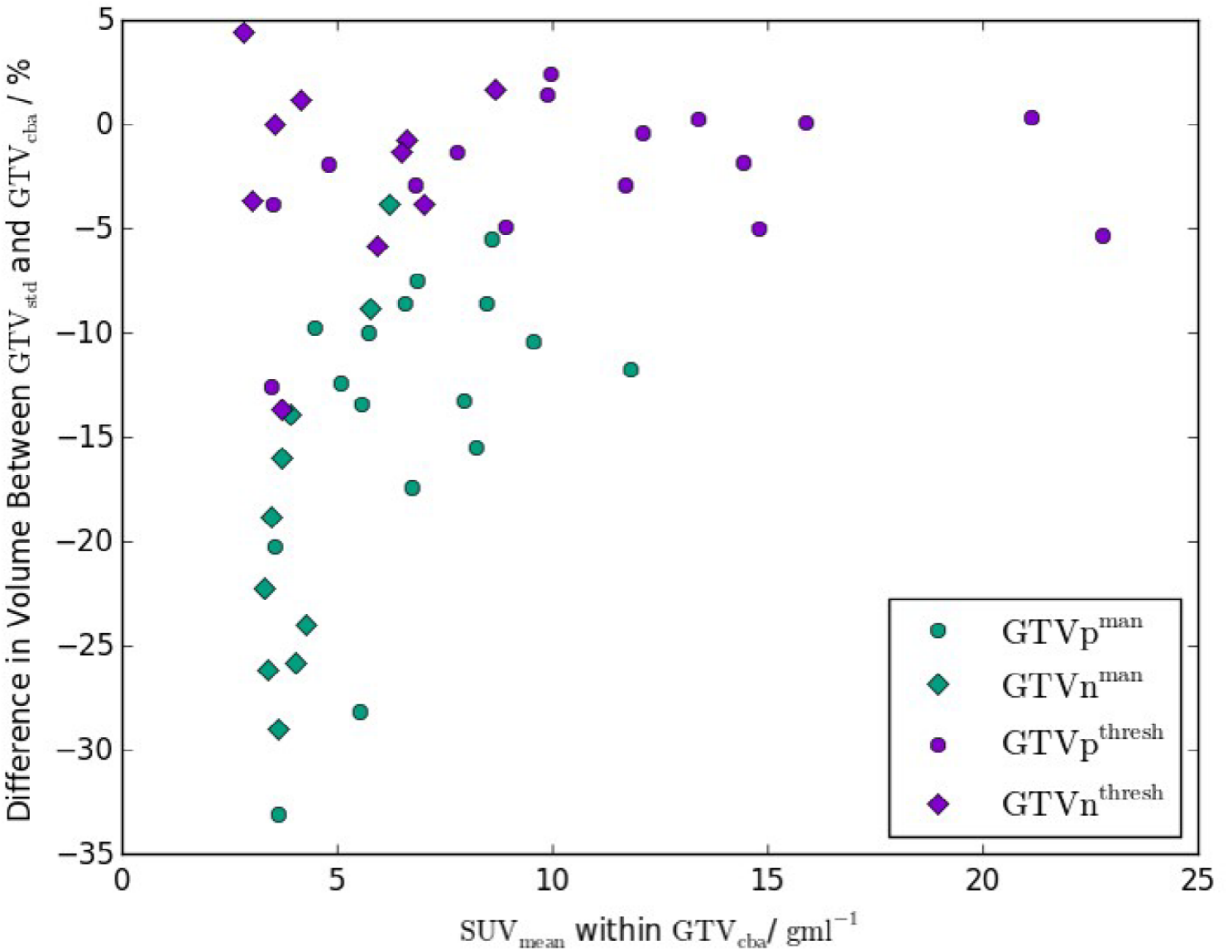
Plot of the difference in GTV volume between ${\mathrm{CTAC}}_{\mathrm{std}}$ to ${\mathrm{CTAC}}_{\mathrm{cba}}$ PET images as a function of the mean SUV within ${\mathrm{GTV}}_{\mathrm{cba}}$ . Both semi‐manual contours (green) and thresholded contours (purple) are shown. Primary GTVs are represented as circles and nodal GTVs as diamonds. GTV, gross tumour volume; PET, positron emission tomography; SUV, standard uptake value.

**FIGURE 6:**
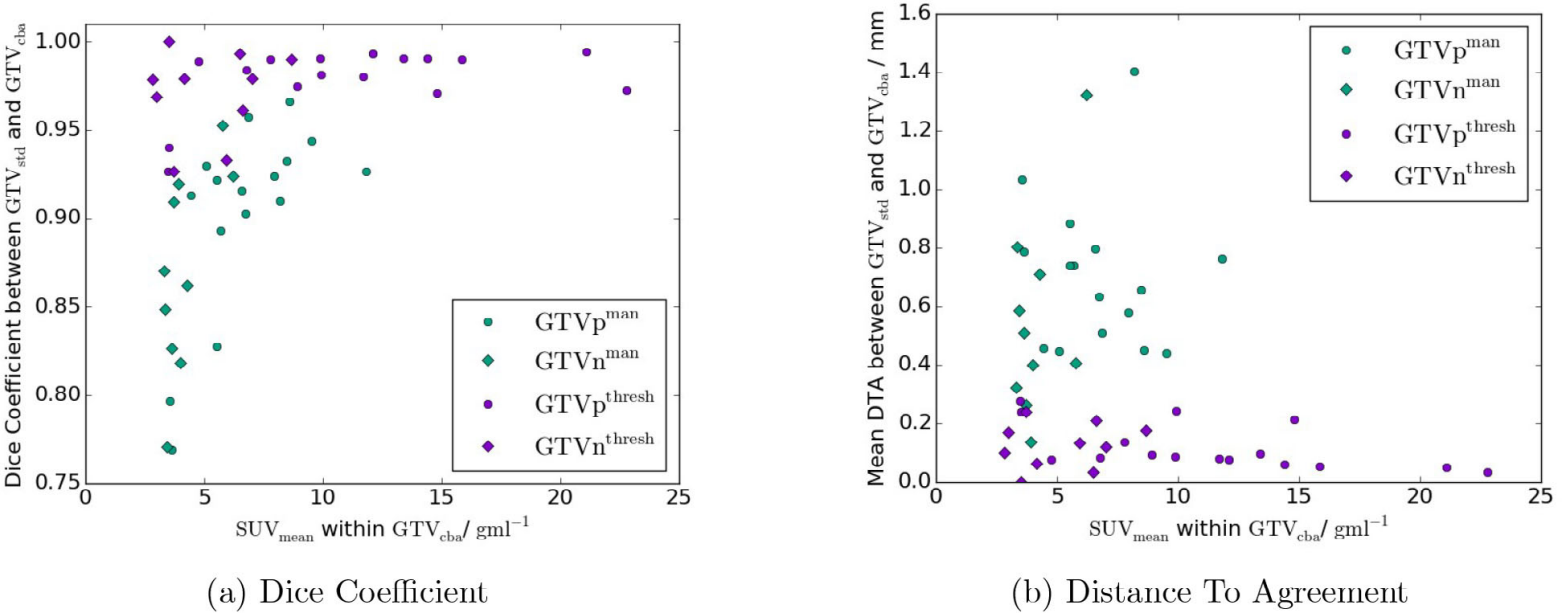
Plot of similarity metrics Dice coefficient (a) and mean distance to agreement (DTA, b) between ${\mathrm{GTV}}_{\mathrm{std}}$ and ${\mathrm{GTV}}_{\mathrm{cba}}$ as a function of ${\mathrm{SUV}}_{\mathrm{mean}}$. Semi‐manual contours (green) and thresholded contours (purple) are shown, with primary GTVs (circles) and nodal GTVs (diamonds) also distinguished. DTA, distance to agreement; GTV, gross tumour volume.

There was a difference in the semi‐manual GTV volumes with the radiotherapy hardware included in the AC map, with the ${\mathrm{GTV}}_{\mathrm{std}}^{\mathrm{man}}$ volumes being −15.9%±1.6% (mean ± standard error, range −33.1%,−3.8%) of the ${\mathrm{GTV}}_{\mathrm{cba}}^{\mathrm{man}}$ volumes. This difference was not statistically significant (p=0.007) over the whole cohort, but appeared to be larger for the less FDG‐avid tumours (see Figure 5). All volume differences greater than 13% occurred in GTVs with ${\mathrm{SUV}}_{\mathrm{mean}}\leq 8.5\;g\;{\mathrm{mL}}^{-1}$. However there remained a reasonable concordance between ${\mathrm{GTV}}_{\mathrm{std}}^{\mathrm{man}}$ and ${\mathrm{GTV}}_{\mathrm{cba}}^{\mathrm{man}}$ with a Dice coefficient of 0.89±0.01 (0.77,0.97) and a mean distance to agreement of 0.65±0.06mm (0.14mm,1.4mm). The Dice coefficient also showed some dependence on ${\mathrm{SUV}}_{\mathrm{mean}}$, although less marked than the volume differences (see Figure 6).

The threshold GTVs were much more similar, with ${\mathrm{GTV}}_{\mathrm{std}}^{\mathrm{thresh}}$ volumes −2.3%±0.8% (−13.7%,4.4%) different to ${\mathrm{GTV}}_{\mathrm{cba}}^{\mathrm{thresh}}$ (p=0.07, Figure 5). Similarly, there was very good concordance between the threshold GTVs, the mean Dice coefficient was 0.98±0.00 (0.93, 1.00) and the mean distance to agreement was 0.12±0.02mm (0.00mm,0.28mm).

### 3.3 Metabolic parameters

**FIGURE 7:**
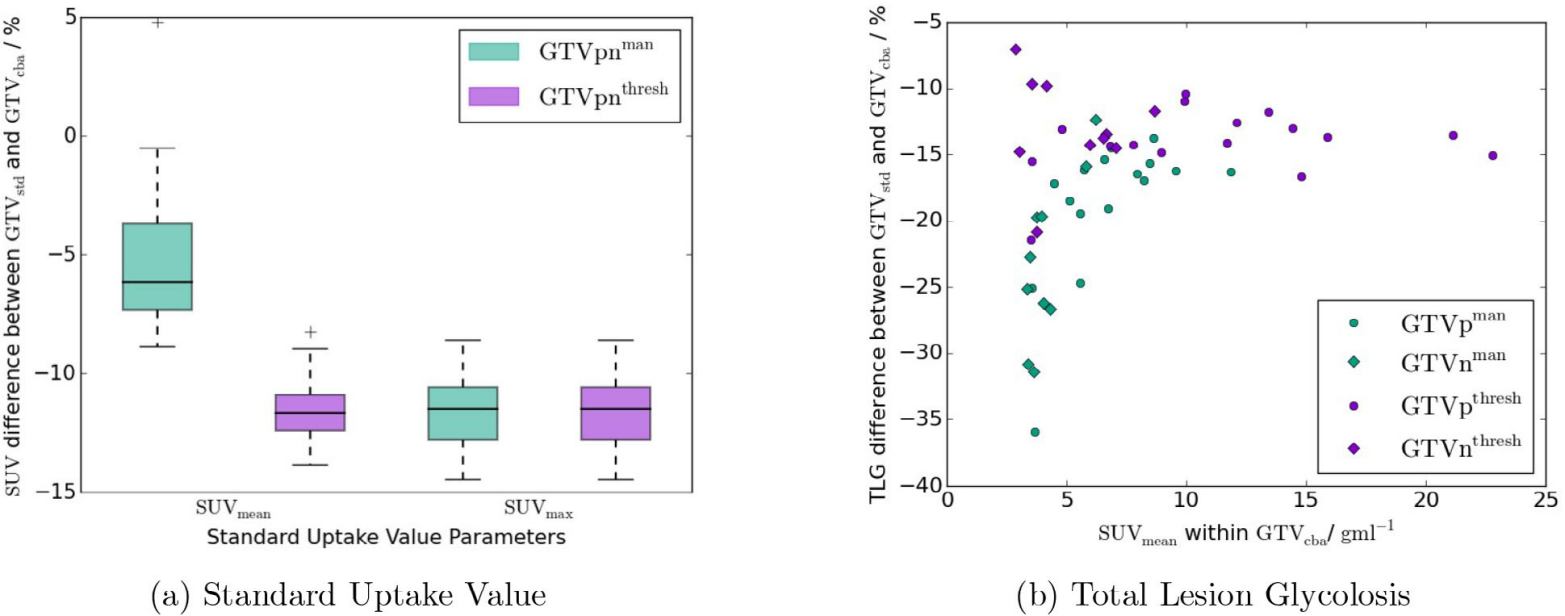
(a) Box plot of the differences in ${\mathrm{SUV}}_{\mathrm{mean}}$ and ${\mathrm{SUV}}_{\max}$ between ${\mathrm{CTAC}}_{\mathrm{std}}$ and ${\mathrm{CTAC}}_{\mathrm{cba}}$ PET images for both primary and nodal GTVs. The rectangles indicate the IQR, with the horizontal black line the median value, the black whiskers the maximum (minimum) data point within Q3+1.5IQR (Q1−1.5IQR) and the black crosses outlier data points. (b) shows the difference in TLG between ${\mathrm{CTAC}}_{\mathrm{std}}$ and ${\mathrm{CTAC}}_{\mathrm{cba}}$ images as a function of ${\mathrm{SUV}}_{\mathrm{mean}}$, with primary GTVs indicated as circles and nodal GTVs diamonds. For both plots semi‐manual GTVs are shown in green and thresholded GTVs in purple. IQR, interquartile range; GTV, gross tumour volume; PET, positron emission tomography; SUV, standard uptake value; TLG, total lesion glycolysis.

There was a substantial drop in the metabolic GTV parameters on the ${\mathrm{CTAC}}_{\mathrm{std}}$ images compared to ${\mathrm{CTAC}}_{\mathrm{cba}}$ images (see Figure 7). The mean percentage difference for the semi‐manual contours of ${\mathrm{SUV}}_{\max}$ was −11.5%±0.3% (−14.5%,−8.6%), ${\mathrm{SUV}}_{\mathrm{mean}}$ was −5.2%±0.6% (−8.9%, 4.8%) both with p<0.001, and TLG was −20.5%±1.2% (−35.9%,−12.4%, p=0.005). The equivalent values for the threshold contours were also significant with ${\mathrm{SUV}}_{\max}$ being −11.5%±0.3% (−14.5%,−8.6%, p<0.001), ${\mathrm{SUV}}_{\mathrm{mean}}$ −11.6%±0.3% (−13.8%,−8.2%, p<0.001) and TLG −13.7%±0.6% (−21.4%,−7.1%, p=0.003).

Comparing the calculated TLG values to the TLG cut‐off values gave 2/8 (using the Ogawa et al. figure) or 5/8 (Choi et al. figure) rectum cancer patients in the poorer prognosis group. Importantly, one patient changed from the good prognosis to poor prognosis group when TLG was calculated using ${\mathrm{CTAC}}_{\mathrm{cba}}$ rather than ${\mathrm{CTAC}}_{\mathrm{std}}$, using the Choi et al. cut‐off value. This patient was not the patient who received the lower activity injection.

## 4 DISCUSSION

PET‐MR imaging has the potential to improve GTV delineation as well as enable dose painting and dose escalation treatment strategies for ano‐rectal radiotherapy. This study aimed to apply a previously developed AC method, which had demonstrated substantial reductions in PET activity loss in phantoms, to PET‐MR images in the radiotherapy position of ano‐rectal cancer patients. In particular, the study aimed to assess the impact of not using this AC method on GTV delineation and GTV metabolic parameter accuracy, to see if using this AC method was required for radiotherapy PET‐MR.

The impact on semi‐manual GTV delineation was mixed. Although the volume difference was not statistically significantly, it was approaching statistical significance (p=0.007) and Bonferonni correction is known to be conservative when the variables being tested are not independent.^[^ ^18^ ^]^ The mean volume difference was −15.9%±1.6%, with differences up to −33.1% for the less FDG‐avid lesions. This suggests it is likely that the volume difference would become statistically significant with a larger number of patients, and therefore that there is an impact on semi‐manual GTV delineation to using ${\mathrm{CTAC}}_{\mathrm{cba}}$. This impact would depend on the method of contouring used and other methods not using a fixed threshold as the basis for delineation could see different impacts when using ${\mathrm{CTAC}}_{\mathrm{cba}}$. However, despite the large volume differences in some patients, the similarity metrics still showed good ageement for most patients (Figure 6). The mean levels of agreement were similar to or better than inter‐observer variability in GTV delineation in rectal cancer patients reported in the literature. Patel et al. reported PET‐CT delineated primary GTVs had Dice coefficients of 0.81±0.03 (mean ± standard error) and nodal GTVs 0.70±0.12.^[^ ^19^ ^]^ Buijsen et al. reported higher Dice coefficients, 0.90 for manual delineations and 0.96 for an automatic delineation using a source‐to‐background ratio method, also for rectal GTVs.^[^ ^20^ ^]^ This suggests that using ${\mathrm{CTAC}}_{\mathrm{std}}$ compared to ${\mathrm{CTAC}}_{\mathrm{cba}}$ introduces differences in manual GTV delineation that are similar to those introduced by inter‐observer variability, although it is important to note that these studies did not use the fixed threshold semi‐manual method of GTV delineation used here. To the best of the author's knowledge, no study has investigated the impact on ano‐rectal target delineation using PET images acquired in the pelvic radiotherapy position.

There was a marked dependence on mean SUV for the volume differences, with much larger differences for the less FDG‐avid lesions (Figure 5). This was probably due to the shallower gradients in SUV around the lower ${\mathrm{SUV}}_{\mathrm{mean}}$ GTVs meaning an 13.8% shift in SUV from using ${\mathrm{CTAC}}_{\mathrm{cba}}$ resulted in a larger volume expansion than in the more FDG‐avid lesions. The Dice coefficient showed a similar if less pronounced trend with ${\mathrm{SUV}}_{\mathrm{mean}}$, with all values <0.90 occuring for GTVs with ${\mathrm{SUV}}_{\mathrm{mean}}<6\;g\;{\mathrm{mL}}^{-1}$. This implies that using ${\mathrm{CTAC}}_{\mathrm{cba}}$ is likely to be more important in GTV delineation for less FDG‐avid lesions. All except one of the semi‐manual nodal GTVs had ${\mathrm{SUV}}_{\mathrm{mean}}<6\;g\;{\mathrm{mL}}^{-1}$ so nodal delineations may require accurate AC to avoid under‐segmentation.

The impact on the thresholded GTV delineations of using ${\mathrm{CTAC}}_{\mathrm{cba}}$ was much less than on the semi‐manual delineations, with small volume differences and high similarity metric scores. This was likely due to the fact that the threshold SUV was a relative value (40% of ${\mathrm{SUV}}_{\max}$), and so the ∼13% shift in SUV changed both ${\mathrm{SUV}}_{\max}$ and the boundary voxels by approximately the same amount, resulting in a very similar volume. In contrast, the semi‐manual delineation used a fixed $SUV=2.5\;g\;{\mathrm{mL}}^{-1}$ threshold as the starting point for delineation, which means the increase in SUVs from using ${\mathrm{CTAC}}_{\mathrm{cba}}$ resulted in a larger volume delineated. There was a large difference between semi‐manual and threshold contours in GTV volume on the same image set, with mean volumes of $44.3\;{\mathrm{cm}}^{3}$ compared to $18.9\;{\mathrm{cm}}^{3}$. This is consistent with a previous study of 18 ano‐rectal cancer patients in PET‐CT images, which found mean semi‐manual volumes of $42.4\pm 6.8\;{\mathrm{cm}}^{3}$ (± standard error) and percentage threshold volumes $15.5\pm 2.9\;{\mathrm{cm}}^{3}$.^[^ ^8^ ^]^ The semi‐manual volumes were closer to the gold standard expert consensus volumes, $36.2\pm 7.2\;{\mathrm{cm}}^{3}$.

There was a much bigger impact from using ${\mathrm{CTAC}}_{\mathrm{cba}}$ rather than ${\mathrm{CTAC}}_{\mathrm{std}}$ on the metabolic parameters. There were statistically significant differences in ${\mathrm{SUV}}_{\mathrm{mean}}$, ${\mathrm{SUV}}_{\max}$ and TLG for both semi‐manual and thresholded GTVs. The differences in ${\mathrm{SUV}}_{\mathrm{mean}}$ for the thresholded volumes and ${\mathrm{SUV}}_{\max}$ for both semi‐manual and thresholded volumes were very similar to each other, with median differences similar to the −13.8% mean per‐pixel SUV difference. The differences in ${\mathrm{SUV}}_{\mathrm{mean}}$ for the semi‐manual volumes were smaller and more variable. This was likely due to the changes in semi‐manual GTV volume with the two ACs, with the larger volumes on the ${\mathrm{CTAC}}_{\mathrm{cba}}$ images lowering the ${\mathrm{SUV}}_{\mathrm{mean}}$ and so partially offsetting the 13.8% increase in per‐pixel SUVs. One GTV actually had a larger ${\mathrm{SUV}}_{\mathrm{mean}}$ in the ${\mathrm{CTAC}}_{\mathrm{std}}$ than the ${\mathrm{CTAC}}_{\mathrm{cba}}$. Examination of this volume indicated that the ${\mathrm{GTV}}_{\mathrm{cba}}^{\mathrm{man}}$ extended over two more axial slices than ${\mathrm{GTV}}_{\mathrm{std}}^{\mathrm{man}}$. This meant ${\mathrm{GTV}}_{\mathrm{cba}}^{\mathrm{man}}$ included more lower SUV pixels, which reduced the ${\mathrm{SUV}}_{\mathrm{mean}}$ to 5% less than ${\mathrm{GTV}}_{\mathrm{std}}^{\mathrm{man}}$, even though the ${\mathrm{SUV}}_{\max}$ in ${\mathrm{GTV}}_{\mathrm{cba}}^{\mathrm{man}}$ was 10% higher than in ${\mathrm{GTV}}_{\mathrm{std}}^{\mathrm{man}}$. This may also be in part due to the difficulty in identifying the primary tumour due to physiological uptake at the adjacent bowel. TLG was also significantly lower on the ${\mathrm{CTAC}}_{\mathrm{std}}$ images, with a similar dependence on ${\mathrm{SUV}}_{\mathrm{mean}}$ as the volume differences.

The clinical significance of these these statistically significant differences in metabolic parameters was difficult to determine. PET‐CT SUV measurements have indicated a test‐retest repeatability of 10%−12% in tumour SUVs when performed under carefully controlled conditions in a research setting.^[^ ^21^ ^]^ In clinical diagnostic settings variability in SUVs is likely to be 15%−20%.^[^ ^22^ ^]^ This is a similar order of variability as the error in SUVs reported in this study. However, the repeatability was determined using gold standard CT AC, and so failing to include the radiotherapy hardware in the AC map would generate an additional, systematic, bias to the SUV measurements. In addition, the SUV accuracy requirements for using SUV measurements for radiotherapy dose painting or treatment response assessment are higher than for routine clinical diagnostic purposes, suggesting the differences in metabolic parameters may be even more clinically significant in this context.^[^ ^23^ ^]^

One way of investigating this is considering the use of PET metabolic parameters for treatment prognosis. This is a pre‐cursor to using SUVs for dose painting or response assessment. Several studies have provided evidence that TLG measured in a pre‐treatment 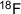
‐FDG‐PET scan are independent prognostic factors for disease‐free and overall survival in rectal cancer patients.^[^ ^5^, ^10^ ^]^ Using the the TLG cut‐off value of Choi et al., 1/8 rectal cancer patients changed prognosis group when SUVs were calculated using ${\mathrm{CTAC}}_{\mathrm{cba}}$ instead of ${\mathrm{CTAC}}_{\mathrm{std}}$. If these prognostic factors are used to guide radiotherapy dose prescriptions, this indicates that acquiring accurate PET images which account for the attenuation of the radiotherapy hardware could be critical.

Only one study has assessed the impact on metabolic parameters when acquiring PET‐MR images in the body radiotherapy position. Paulus et al. evaluated differences in three lung cancer patients scanned on a Siemens PET‐MR scanner.^[^ ^24^ ^]^ Images were acquired with and without the anterior array coil on a coil bridge but with the flat couch top in both cases. The differences in ${\mathrm{SUV}}_{\mathrm{mean}}$ and ${\mathrm{SUV}}_{\max}$ between no coil and bridge, and coil and bridge images, without AC, was −10.0%±2.4% and −11.1%±2.0% respectively. Including AC of the anterior coil and coil bridge reduced this to −2.4%±3.3% (${\mathrm{SUV}}_{\mathrm{mean}}$) and −3.9%±2.6% (${\mathrm{SUV}}_{\max}$). These results are not directly comparable to the results reported in this study because they included the flat couch top in the AC for both images.

A weakness of the methodology presented here is there has been no comparison to a patient acquisition without the radiotherapy hardware and anterior coil present. The method has been evaluated previously on phantoms, demonstrating that SUV measurements with the hardware included in the AC map were significantly closer to the gold standard than without.^[^ ^15^ ^]^ Therefore it is reasonable to assume the same applies in patients. A patient PET acquisition without the radiotherapy hardware would have provided a gold standard PET image to confirm this phantom result. However, this would also have introduced several confounding variables between the images in the radiotherapy and gold standard setups. These would include differences due to difficulties in registering patient images acquired in different setups and differences in SUV distribution due to imaging at a different time‐point. In addition, it would have added significant imaging time for patients. Therefore it was decided to compare AC maps with and without the radiotherapy hardware included to assess the impact of changing the AC map and rely on the phantom measurements alone to confirm the accuracy of the AC map with radiotherapy hardware included. The phantom measured difference in activity loss between using the AC map with radiotherapy hardware included was 15.0%,^[^ ^15^ ^]^ which is close to the whole image difference in SUV in this study of 13.8%, suggesting the phantom measurements do translate to patients. Winter et al. did compare PET‐MR images in the radiotherapy position with appropriate AC and diagnostic position for head and neck patients.^[^ ^25^ ^]^ They concluded thresholded GTV contours were equivalent between the diagnostic and radiotherapy images based on a median Dice score of 0.89 and a median average assymetric surface distance of 0.6mm. This confirms it is reasonable to use PET‐MR images in the radiotherapy position with the radiotherapy hardware in the AC map as a gold standard. Interestingly, the agreement in threshold GTV delineation in this study was substantially higher, with mean Dice 0.98 and mean distance to agreement of 0.12mm. This suggests the impact on percentage threshold GTV delineation from not using the radiotherapy hardware in the AC map is minimal.

The other major component of AC for PET‐MR image reconstruction is accounting for the attenuation of the patient. The current vendor supplied method uses a Dixon MR sequence to segment the patient into fat, water and air tissue classes which are then assigned linear attenuation coefficients.^[^ ^26^ ^]^ This has been shown to introduce SUV errors. In this study this problem was avoided by using the registered radiotherapy planning CT image for patient AC. Improved methods for accounting for patient attenuation in PET‐MR images are currently being investigated. From a radiotherapy perspective, algorithms used to generate synthetic CTs from MR images for MR‐only radiotherapy are in clinical use.^[^ ^27^, ^28^ ^]^ One of these algorithms has demonstrated improvements in patient PET AC compared to the previous Dixon‐based method,^[^ ^29^ ^]^ although the magnitude of the difference in SUVs was less than half of the discrepancy reported here. This suggests that incorporating the radiotherapy hardware is more important for accurate PET quantification than accurate patient AC. Future work could investigate combining synthetic CT patient AC with AC of the radiotherapy hardware evaluated in this study.

## 5 CONCLUSION

Acquiring PET‐MR images for radiotherapy planning requires patients to be imaged in the radiotherapy position on a flat couch with a coil bridge. Applying AC maps that incorporate this hardware and the MR anterior coil was feasible in radiotherapy patients and resulted in a 13.8% increase in SUVs. This resulted in differences in GTV delineation which were approaching statistical significance, and were more pronounced for less FDG‐avid volumes. Evaluating this in a larger patient cohort is likely to provide a more conclusive result. It also had large and significant differences in metabolic measurements, which could have clinically significant consequences. This suggests that it is likely to be beneficial to incorporate AC of the radiotherapy hardware and anterior coil for radiotherapy planning PET‐MR images in the pelvis, especially if used for dose painting and treatment prognostication.

## AUTHOR CONTRIBUTIONS

Jonathan J. Wyatt: Contributed to the study design, data acquisition, data anlysis and drafted the manuscript. George Petrides: Contributed to the study design, data acquisition and revised the manuscript. Rachel A. Pearson: Contributed to the study design, data acquisition and revised the manuscript. Hazel M. McCallum: Contributed to the study design, data analysis and revised the manuscript. Ross J. Maxwell: Contributed to the study design, data analysis and revised the manuscript.

## CONFLICT OF INTEREST STATEMENT

G.P. declares that he receives fees from GE Healthcare for reporting DaTSCANs and honoraria for DaTSCAN educational presentations. J.J.W. declares receiving an honorarium from GE Healthcare for speaking in a webinar series. None of these had an impact on the work reported in this study.
